# The effects of a high dosage of creatine and caffeine supplementation on the lean body mass composition of rats submitted to vertical jumping training

**DOI:** 10.1186/1550-2783-8-3

**Published:** 2011-03-01

**Authors:** Frederico SC Franco, Neuza MB Costa, Susana A Ferreira, Miguel A Carneiro-Junior, Antônio J Natali

**Affiliations:** 1 Federal Institute for Education, Sciences and Technology, Rio Pomba-MG, Brazil; Av. Dr. Sebastião da Paixão s/n; Rio Pomba-MG; Postal code: 36180-000; Brasil; 2 Department of Nutrition and Health, Federal University of Viçosa, Av. Peter Henry Rolfs s/n; Viçosa-MG; Postal code: 36.570-000; Brazil; 3 Department of Physical Education, Federal University of Viçosa, Av. Peter Henry Rolfs s/n; Viçosa-MG; Postal code: 36.570-000; Brazil

## Abstract

**Background:**

The influences of creatine and caffeine supplementation associated with power exercise on lean body mass (LBM) composition are not clear. The purpose of this research was to determine whether supplementation with high doses of creatine and caffeine, either solely or combined, affects the LBM composition of rats submitted to vertical jumping training.

**Methods:**

Male Wistar rats were randomly divided into 8 groups: Sedentary (S) or Exercised (E) [placebo (Pl), creatine (Cr), caffeine (Caf) or creatine plus caffeine (CrCaf)]. The supplemented groups received creatine [load: 0.430 g/kg of body weight (BW) for 7 days; and maintenance: 0.143 g/kg of BW for 35 days], caffeine (15 mg/kg of BW for 42 days) or creatine plus caffeine. The exercised groups underwent a vertical jump training regime (load: 20 - 50% of BW, 4 sets of 10 jumps interspersed with 1 min resting intervals), 5 days/wk, for 6 weeks. LBM composition was evaluated by portions of water, protein and fat in the rat carcass. Data were submitted to ANOVA followed by the Tukey post hoc test and Student's *t *test.

**Results:**

Exercised animals presented a lower carcass weight (10.9%; *P *= 0.01), as compared to sedentary animals. However, no effect of supplementation was observed on carcass weight (P > 0.05). There were no significant differences among the groups (P > 0.05) for percentage of water in the carcass. The percentage of fat in the group SCr was higher than in the groups SCaf and ECr (P < 0.05). A higher percentage of protein was observed in the groups EPl and ECaf when compared to the groups SPl and SCaf (P < 0.001). The percentage of fat in the carcass decreased (P < 0.001), while those of water and protein increased (P < 0.05) in exercised animals, compared to sedentary animals. Caffeine groups presented reduced percentage of fat when compared to creatine supplemented groups (P < 0.05).

**Conclusions:**

High combined doses of creatine and caffeine does not affect the LBM composition of either sedentary or exercised rats, however, caffeine supplementation alone reduces the percentage of fat. Vertical jumping training increases the percentages of water and protein and reduces the fat percentage in rats.

## Background

Creatine and caffeine are among the main ergogenic agents used in sports aiming to achieve increased power, performance, lean body mass (LBM) and delayed fatigue [1-5].

Creatine supplementation has been associated with increased LBM and strength [2,5,6] and reduced muscle mass loss [7]. Combined with power exercise, creatine supplementation may improve performance by spearing muscle glycogen, slowing down phosphocreatine dynamics in exercise and subsequent recovery and accelerating recovery between sets of exercise [8-11], which subsequently may allow a greater number of exercise bouts to be performed. Thus, it may potentiate the strength exercise effects and result in increased LBM in humans and animals [11,12]. However, its pharmacokinetics may by influenced by dietary components, such as caffeine and bicarbonate [13].

Caffeine ingestion enhances power output during high-intensity cycling in humans [14,15]. Caffeine is known to act directly on skeletal muscle leading to increased transmission of neural stimulus to the neuron-muscular junction [16]. It also blocks the central nervous system adenosine receptors [1] and delay fatigue during power exercise in humans [16] and animals [1,17]. These caffeine effects could enhance power training performance and hence promote alterations in body composition [18]. Nevertheless, the potential of chronic caffeine ingestion to enhance muscular strength and LBM has not been explored. Studies on the effects of acute caffeine ingestion on muscular strength have provided divergent data. For example, while a study by Jacobson et al. [19] demonstrated that a 7 mg/kg caffeine dose significantly enhanced muscular strength, Astorino et al. [20] found no effect of a 6 mg/kg dose on humans.

Although a pre-workout supplement containing caffeine, creatine and amino acids combined with three weeks of high-intensity interval training increased the LBM in humans [21], the combined ingestion of creatine and caffeine may eliminate the ergogenic action of creatine supplementation, which is the increase in muscular stocks and exercise performance during intense intermittent exercise [13,22,23]. However, caffeine was found to be ergogenic when taken six days after creatine ingestion or caffeine abstinence [24]. While creatine increased muscle phosphocreatine level and shortened muscle one-half relaxation time in rats [25], short term caffeine intake inhibited muscle relaxation [22]. This negative impact of caffeine on relaxation time contributes to counteract the beneficial effect of creatine supplementation on exercise training performance, which might affect the LBM composition.

Thus, the present study was carried to investigate the current uncertainties about the influence of creatine and caffeine associated with power exercise on the LBM composition and on the counteraction of these ergogenic agents. We also considered that the consumption of supplements in excessive doses might expose users to serious side effects [26,27], and that studies on human body composition are carried out using indirect measurements of the LBM [5,11,28,29]. Thus, by using direct measurement of the LBM composition on a rat model, the purpose of this study was to determine whether high doses of caffeine and creatine supplementation, either solely or combined, affect the LBM composition of rats submitted to a power training regime based on a model of intermittent vertical jumps.

## Methods

### Animals and experimental procedures

Seven-week-old male Wistar rats, weighing 142.7 ± 10.46 g at the onset of the experiment, were kept on a normal light/dark cycle in a climate-controlled environment throughout the study. The animals were maintained in individual cages and were unable to perform spontaneous exercise. Then, they were randomly assigned to one of the following groups (n = 10): SPL (Sedentary placebo); SCr (Sedentary creatine); SCaf (Sedentary caffeine); SCrCaf (Sedentary creatine plus caffeine; EPL (Exercised placebo); ECr (Exercised creatine); ECaf (Exercised caffeine) or ECrCaf (Exercised creatine plus caffeine, n = 09). The animals were pair-fed (15 to 20 g/day) AIN-93 M powdered diet, as recommended [30], and received distilled water *ad libitum*. The principles of laboratory animal care (NIH publication No. 86-23, revised 1985) were followed, as well as the specific national laws (n° 9.605/1998). All procedures were approved by the Ethics Committee of the Federal University of Viçosa, Brazil.

### Creatine and caffeine supplementation

Every day, the animals from the groups SCr and ECr were supplemented with creatine, while those from the groups SCrCaf and ECrCaf received creatine plus caffeine. Creatine was given using a two-stage procedure: loading and maintenance. During the loading stage (7 days), in the first week, a dosage of 0.430 g of powdered creatine monohydrate (Sigma) per kg of body weight per day was added to 15 g of the diet (AIN-93 M powdered diet) and given to the groups SCr, ECr, SCrCaf and ECrCaf. The maintenance stage lasted 5 weeks, starting from the second week, and a dosage of 0.143 g of creatine/kg body weight/day was added to 15 g of the diet and given to the groups SCr, SCrCaf, ECr and ECrCaf. From the second to the sixth week, a dosage of 10 mg of powdered caffeine (Sigma) per kg body weight per day was given to animals from the groups SCaf, SCrCaf, ECaf and ECrCaf. Animals from the groups SPl and EPl received diet only. From the fourth week on, all animals received 20 g of the diet every day.

### Exercise training protocol

During the first week, the animals from the groups EPl, ECr, ECaf and ECrCaf swam for 30 min/day in a tank (60 cm wide, 75 cm long, 80 cm deep) filled with water at 32 ± 1°C to adapt to the environment. The exercise training regime comprised vertical jumps from the bottom of the tank to the surface water. To augment the exercise intensity, an external load (% of body weight) was added to the animal by using plumber spheres in a lycra vest. The deepness of water was determined by an average percentage of the animals' length (i.e. distance between the end of the posterior members and the nostril) (Table 1). The training program was conducted from the second to the sixth week of the experiment and the animals performed 4 sets of 10 jumps with 1 minute recovery time between sets, 5 days/week (Table 1). This exercise training regime and the working apparatus are currently used in our laboratory and elsewhere [31]. During the last training session, concentrations of blood lactate of the exercised animals were monitored in three moments. Blood samples (25 μL) were obtained from the tail vein at rest, after the 2^nd ^and 4^th ^sets of jumps and the lactate concentrations were measured (Accusport BM-Lactate, Roche Diagnostics, Mannheim, Germany).

**Table 1:** Exercise training program schedule.

Week	Sets × repetitions	Load (% rat body weight)	Water level (% rat length)
1^st ^(adaptation)	30 min	0	80
2^nd^	4 × 10	20-25	120
3^rd^	4 × 10	30-35	130
4^th^	4 × 10	40	140
5^th^	4 × 10	45	145
6^th^	4 × 10	50	150

### Body composition

After the treatments, the animals were euthanized (CO_2_). Their skin and viscera were separated from muscles and bones (empty carcass) and head and tail were disposed. The empty carcass was weighed and stored in a freezer (-20°C) for subsequent analyses. Body water percentage was evaluated using the gravimetric method by evaporation of water in an oven (Fanem, Guarulhos - SP, Brazil) at 105°C for 24 h. Fat percentage was determined by the gravimetric process in a Soxhlet equipment, with the use of ethylic ether as solvent for the 8-hour extraction. Protein percentage was calculated by the indirect method of nitrogen determination [Protein (g) = nitrogen (g) × 6.25] and the Kjeldahl method [32].

### Urinary creatinine content

Urine samples were collected during a 24 h-period at the end of the first, second and sixth weeks of the experiment. Urinary creatinine was determined through automatic UV/VIS spectrophotometry (ALIZÉ^® ^equipment, Biomêrieux - France) using commercial kits.

### Statistical analysis

All data were submitted to the normality test (Kolmogorov-Smirnov). ANOVA was once used to compare body weight, carcass weight and percentages of water, fat and protein, and urinary creatinine among the groups and supplementation factor effects. Whenever a significant F-value was obtained, a post-hoc test with a Tukey adjustment was performed for multiple comparison purposes. The exercise factor effect (sedentary vs. exercised groups) was determined by the Student's *t *test. All data analyses were performed using the Sigma Stat 3.0 software system (SPSS, Illinois - Chicago, USA) and the statistical significance was set at *P *< 0.05.

## Results

The concentrations of blood lactate increased similarly in all exercised animals (ANOVA One-Way Repeated Measures, *P *< 0.05) from rest (2.7±0.6 mmol/L; mean ± SD), to the second set (6.9 ± 1.4 mmol/L) and fourth set (9.2 ± 1.8 mmol/L) of vertical jumping moments.

### Lean body mass composition

Food intake was controlled to 15 to 20 g/day, according to the age and consumption of the animals. No difference in food intake was observed among the groups throughout the experimental period (data not shown). The initial body weights of the animals were not different (P > 0.05) among the groups (Table 2). By the end of the experimental period, the groups SPl and SCaf exhibited higher body weights compared to EPl and ECaf, respectively (Table 2). The exercised animals presented a lower body weight (11.6%; *P *= 0.001), compared to the sedentary animals. The carcass weight was higher in SPl and SCaf, compared to the groups EPl and ECaf (P = 0.034 and P < 0.01; respectively). Likewise, the exercised animals presented a lower carcass weight (10.9%; *P *= 0.01), as compared to the sedentary animals. However, no effect of supplementation was observed either in body weight or carcass weight (P > 0.05).

**Table 2:** Body and carcass weights.

Groups	Initial BW (g)	Final BW (g)	Carcass weight (g)
SPl (n = 10)	141,9 ± 8,4	314.0 ± 7.7^a^	147.7 ± 6.6

SCr (n = 10)	140,1 ± 9,9	306.6 ± 16.0^a ^	142.9 ± 8.3

SCaf (n = 10)	142,8 ± 9,8	327.2 ± 8.2^a^	154.5 ± 6.0

SCrCaf (n = 09)	145,0 ± 9,4	307.6 ± 15.2^a ^	140.5 ± 8.8

EPl (n = 09)	139,9 ± 13,3	284.8 ± 9.7^ab^	132.9 ± 6.5^b^

ECr (n = 07)	141,0 ± 13,2	286.7 ± 20.8^a^	134.7 ± 10.6

ECaf (n = 08)	146,8 ± 9,4	264.6 ± 15.5^ac^	126.3 ± 16.5^c^

ECrCaf (n = 09)	144,1 ± 12,7	275.2 ± 26.3^a^	128.3 ± 12.8

**Exercise factor**			

Sedentary	-	314.0 ± 14.5	146.5 ± 9.0

Exercised	-	277.7 ± 27.8^d^	130.4 ± 12.0^d^

**Supplementation factor**			

Placebo (EPl+SPl)	-	300.2 ± 17.2	140.7 ± 9.9

Creatine (ECr+SCr)	-	298.4 ± 20.2	139.5 ± 9.9

Caffeine (ECaf+SCaf)	-	299.4 ± 43.0	142.0 ± 18.4

Creatine+Caffeine (ECrCaf+SCrCaf)	-	291.4 ± 26.7	134.4 ± 12.4

Data are mean ± SD. n, number of animals. Statistical significance (*P *< 0.05): ^a ^*vs*. initial BW; ^b ^*vs*. SPl; ^c ^*vs*. SCaf; ^d ^*vs*. Sedentary for the same column. BW, body weight. SPl, sedentary placebo. SCr, sedentary creatine. SCaf, sedentary caffeine. SCrCaf, sedentary creatine plus caffeine. EPl, exercised placebo. ECr, exercised creatine. ECaf, exercised caffeine. ECrCaf, exercised creatine plus caffeine.

**Figure 1:**
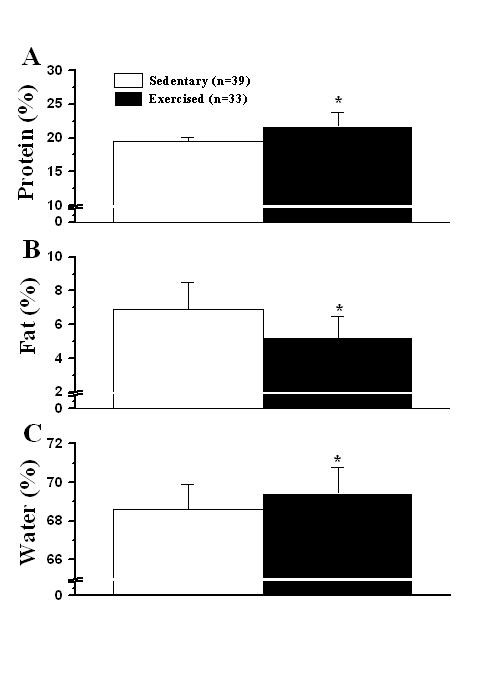
**Lean body mass composition and the exercise factor**. (A) percentage of protein, (B) percentage of fat, (C) percentage o water. Data are mean ± SD (% of carcass weight, independent of supplementation). n, number of animals. *, denotes significant differences from sedentary animals (*P *< 0.05).

Data of carcass content (protein, fat and water) are presented as percentage of carcass weight. There were no significant differences among groups (P > 0.05) for percentage of water (data not shown). The percentage of fat in the group SCr (7.8 ± 1.8%) was higher than that in the groups SCaf (5.8 ± 1.3%) and ECr (5.6 ± 1.5%) (P = 0.039 and P = 0.043, respectively). Besides, it was observed a higher percentage of protein in the groups EPl (21.5 ± 0.6%) and ECaf (22.8 ± 3.0%) when compared to SPl (19.5 ± 0.7%) and SCaf (19.6 ± 0.4%; P < 0.001). With respect to exercise, it was observed a decreased percentage of fat in carcass (Figure 1B; P < 0.001) and increased water (Figure 1C; P = 0.021) and protein percentages (Figure 1A; P < 0.001) in exercised animals, as compared to sedentary animals, independent of supplementation.

**Figure 2:**
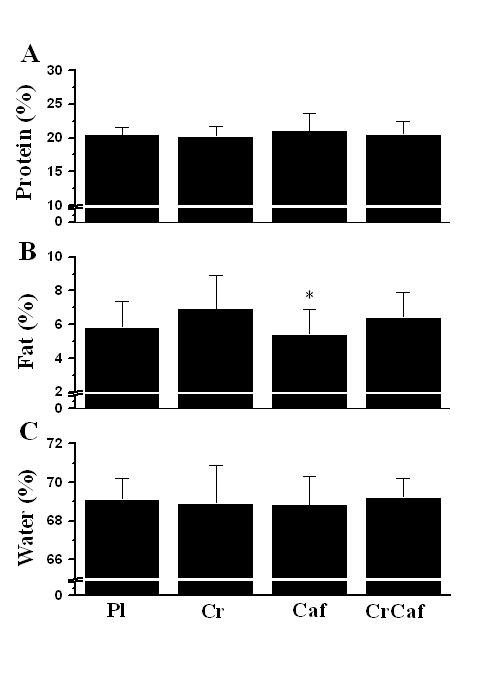
**Lean body mass composition and the supplementation factor**. (A) percentage of protein, (B) percentage of fat, (C) percentage o water. Data are mean ± SD (% of carcass weight, independent of exercise). Pl, placebo (n = 19 animals). Cr, creatine (n = 17 animals). Caf, caffeine (n = 18 animals). CrCaf, creatine plus caffeine (n = 18 animals). *, denotes significant difference from Cr groups (*P *< 0.05).

Regarding the supplementation factor, it was observed that caffeine groups presented reduced percentage of fat in the carcass, as compared to creatine groups (Figure 2B; P = 0.038), independent of exercise. No effects of supplementation were observed on the protein and water percentages (Figure 2A and 2C).

### Urinary creatinine

It was observed a positive correlation between body weight and urinary creatinine (Pearson, r = 0.402 and P < 0.001). Therefore, the urinary creatinine data were normalized by the body weight of the animals and presented as urinary creatinine to body weight ratio (mg/24 h·g) (Table 3). During the first week, urinary creatinine was not different (P > 0.05) among the groups and was affected by neither exercise nor supplementation factors.

**Table 3:** Urinary creatinine.

Groups	1^st ^Week (mg/24 h.g)	2^nd ^Week (mg/24 h.g)	6^th ^Week (mg/24 h.g)
SPl (n = 10)	0.243 ± 0.082	0.217 ± 0.034^a^	0.240 ± 0.047
SCr (n = 10)	0.226 ± 0.038	0.284 ± 0.033^A^	0.255 ± 0.036
SCaf (n = 10)	0.234 ± 0.027	0.208 ± 0.030^a^	0.211 ± 0.030
SCrCaf (n = 09)	0.242 ± 0.020	0.245 ± 0.060	0.234 ± 0.011
EPl (n = 09)	0.231 ± 0.023	0.223 ± 0.040^c^	0.223 ± 0.018
ECr (n = 07)	0.240 ± 0.050	0.301 ± 0.044^A^	0.252 ± 0.015^Bd^
ECaf (n = 08)	0.226 ± 0.023	0.208 ± 0.027^c^	0.204 ± 0.021
ECrCaf (n = 09)	0.259 ± 0.014	0.288 ± 0.051^bd^	0.263 ± 0.026^d^

**Exercise Factor**			

Sedentary (n = 39)	0.236 ± 0.046	0.238 ± 0.049	0.235 ± 0.040
Exercised (n = 33)	0.240 ± 0.032	0.258 ± 0.057	0.236 ± 0.030^B^

**Supplementation Factor**			

Placebo (n = 19)	0.236 ± 0.058	0.220 ± 0.036	0.232 ± 0.036
Creatine (n = 17)	0.233 ± 0.044	0.293 ± 0.039^Aef^	0.253 ± 0.027^Bf^
Caffeine (n = 18)	0.231 ± 0.025	0.208 ± 0.028^A^	0.207 ± 0.026^A^
Creatine+Caffeine (n = 18)	0.250 ± 0.025	0.267 ± 0.059^ef^	0.248 ± 0.033^f^

Data are mean ± SD. n, number of animals. Statistical significance (*P *< 0.05): ^A ^*vs*. 1^st ^week; ^B ^*vs*. 2^nd ^week (ANOVA Repeated Measures) for the same line. ^a ^*vs*. SCr; ^b ^*vs*. EPl; ^c ^*vs*. ECr; ^d ^*vs*. ECaf; ^e ^*vs*. Placebo; ^f ^*vs*. Caffeine (Tukey Test) for the same week. SPl, Sedentary placebo. SCr, sedentary creatine. SCaf, sedentary caffeine. SCrCaf, sedentary creatine plus caffeine. EPl, exercised placebo. ECr, exercised creatine. ECaf, exercised caffeine. ECrCaf, exercised creatine plus caffeine.

During the second week, the urinary creatinine level in the group SCr was higher than the level in SPl and SCaf (P = 0.023 and P = 0.005, respectively, Table 3). The group ECr exhibited higher creatinine than EPl and ECaf (P = 0.002 and P < 0.001, respectively). Likewise, ECrCaf creatinine was higher, compared to EPl and ECaf (P = 0.017 and P = 0.003, respectively). However, there was no difference in urinary creatinine between the sedentary and exercised animals. Regarding supplementation, it was observed that creatine and creatine plus caffeine groups increased their creatinine excretion as compared to placebo and caffeine groups (P < 0,001). It was also verified that urinary creatinine in creatine groups was higher and in caffeine groups, lower, compared to the results of the first week (P < 0.05).

During the sixth week, it was observed that urinary creatinine in the groups ECr and ECrCaf was higher than in ECaf (P < 0.001, Table 3). ECr presented higher urinary creatinine when compared to that of the second week (P < 0.05). There were no differences between exercised and sedentary animals in the sixth week. However, the exercised animals presented lower urinary creatinine when compared to those of the second week (P < 0.05). Concerning supplementation, it was verified that the creatine and creatine plus caffeine groups exhibited higher creatinine as compared to the caffeine groups (P < 0.001 and P = 0,001, respectively). In addition, the creatinine levels of the creatine group were lower than those in the second week. The caffeine groups also presented lower creatinine than those in the first week (P < 0.05).

## Discussion

We demonstrated that supplementation with high combined doses of creatine and caffeine did not affect the LBM composition of either sedentary or exercised rats. However, caffeine supplementation alone reduced the percentage of fat in the carcass. In addition, the employed model of power training increased the percentages of water and protein and reduced the fat percentage in rats.

One of the main observations of our study was that animals supplemented with creatine or creatine plus caffeine did not present increased water retention in skeletal muscles (carcass). It is suggested that creatine supplementation leads to intramuscular water accumulation caused by its high osmotic power [7,33]. Our results do not corroborate such hypothesis and are consistent with the similarity of body weight among our experimental groups as an increase in water retention in response to creatine ingestion might have augmented body weight [13,34]. Even though the methods of weighing were indirect, this lack of increase in body weight caused by creatine supplementation has been reported elsewhere [2,11,29].

Despite the fact that caffeine exerts a slight diuretic effect [15], which could have reduced water content, contrasting the effects of creatine [35], our study revealed that caffeine ingestion did not affect the percentage of water in the lean body mass. Similar results were found by Vanakoski et al. [36], although in our experiment, caffeine dosage was 2.14 times higher.

Concerning exercise effects, we observed an increased percentage of water in the carcass of the exercised animals. Although we have not assessed the content of muscle glycogen, it is thought that such effect is associated with the ability of exercise to promote accumulation of muscular glycogen, since 2.7 g of water are incorporated in the muscle per each gram of glycogen incorporated [37]. Our results agree with those reported by Cortright et al. [38].

Our observation that creatine or creatine plus caffeine did not affect the protein percentage of lean body mass demonstrates the absence of differences in body weight among our experimental groups. The increased in muscular mass in response to creatine ingestion [5,11,34,35] may be due to the increased water retention in muscles [13]. However, such effect was not observed in the present study. Similar results were found by others [2,29,39]. When measuring urinary nitrogen, Jowko et al. [40] verified that creatine did not affect the retention of body nitrogen, suggesting that creatine would not increase protein incorporation. In the present study, even though creatine doses were very high, it did not affect protein percentages in the carcass of creatine supplemented groups.

We demonstrated that the exercise training regime employed here increased the percentage of protein in the carcass, despite the reduction in the final body weight. This finding is consistent with those presented in the literature inasmuch as there is a large body of evidences of skeletal and cardiac muscle hypertrophy in response to intermittent power and running exercises in humans and animals [11,12,40].

Our results revealed that high-dose caffeine supplementation reduced the fat percentage of the lean body mass as compared to creatine ingestion, independently of the exercise training. It has not been mentioned the direct effect of creatine on skeletal muscle fat [2,11]. However, the ingestion of caffeine may increase the turnover and mobilization of free fatty acid [22,41,42] and save muscular glycogen storages [22], which would result in reduced body weight [42]. Caffeine intake increases the basal metabolic rate and catecholamine release [41,43]. Caffeine may also inhibit the activity of the phosphodiesterase enzyme, which increases the levels of AMPc and reduces the activity of hormone-sensitive lipase, leading to higher lipolysis [44]. However, we found no differences in body weight among the groups SPl and EPl, as compared to SCaf and ECaf, respectively. We also demonstrated that the group SCaf presented higher body weight than ECaf and that the exercised animals exhibited lower body weight, as compared to the sedentary animals. Therefore, such reduction in the percentage of fat in the carcass of animals supplemented with caffeine may indicate the interference of exercise instead of caffeine ingestion.

We observed that the exercised animals exhibited lower body weight as well as lower fat percentages compared to the sedentary animals. Although in our model of power exercise the main source of energy is the anaerobic glycolysis, oxygen consumption continues high after exercise due to the increased energetic metabolism of active muscles, an effect of post-exercise oxygen consumption (EPOC) [28,45]. Therefore, such reduction in fat percentage might have not been caused by energy consumption during the vertical jump sets, but partly by oxygen deficit and post-exercise energy costs via EPOC. Malatesta et al. [28] demonstrated that lipid oxidation during post exercise recovery increased in response to intermittent and continuous exercise compared with the time-matched no-exercise controls. The lower fat percentage in the lean body mass of the exercised animals might have resulted from their higher protein percentage as compared to the sedentary animals.

In the present study, neither supplementations nor exercise training affected the excretion of urinary creatinine during the first week. In the second week, the creatinine from the groups creatine or creatine plus caffeine was higher than that from the placebo group, and also higher as compared to the first week. On the other hand, urinary creatinine decreased. Thus, the significance of creatine and creatine plus caffeine effects from the second week has disappeared. These results indicate that the ingestion of high doses of creatine (0.431 g·kg) during the load phase promoted increased excretion of urinary creatinine via a non-enzymatic reaction, as demonstrated by other authors [13,29,45]. Our data also suggest that the load phase could be more important in increasing body creatine storages, since after the phase of creatine maintenance (6^th ^week), urinary creatinine excretion was reduced.

Finally, caffeine ingestion did not affect creatinine excretion. Such finding suggests that caffeine ingestion had no effect on creatine pharmacokinetics. However, our data do not allow us to substantiate such suggestion as we did not measure the muscular content of creatine and its clearance. This is a limitation of this study and requests further investigations.

## Conclusion

In conclusion, high combined doses of creatine and caffeine does not affect the LBM composition of either sedentary or exercised rats, however, caffeine supplementation alone reduces the percentage of fat in the carcass. The employed vertical jump regimen increases the percentages of water and protein and reduces the fat percentage in these animals.

## Competing interests

The authors declare that they have no competing interests.

## Authors' contributions

All authors have read and approved the final manuscript. AJN is the principal investigator of the project. FSCF, NMBC and AJN designed the study; FSCF, SAF and MACJ collected the data; FSCF and AJN conducted data analysis; FSCF and AJN wrote the manuscript.
